# *De Novo* Sequencing and Transcriptome Analysis of *Pleurotus eryngii* subsp. *tuoliensis* (Bailinggu) Mycelia in Response to Cold Stimulation

**DOI:** 10.3390/molecules21050560

**Published:** 2016-05-17

**Authors:** Yong-Ping Fu, Yuan Liang, Yue-Ting Dai, Chen-Tao Yang, Ming-Zheng Duan, Zhuo Zhang, Song-Nian Hu, Zhi-Wu Zhang, Yu Li

**Affiliations:** 1 Engineering Research Center of Chinese Ministry of Education for Edible and Medicinal Fungi, College of Agriculture, Jilin Agricultural University, Changchun 130118, China; yongpingfu81@126.com (Y.-P.F.); daiyueting18@163.com (Y.-T.D.); duanmingzheng@hotmail.com (M.-Z.D.); zzo1018@126.com (Z.Z.); 2 Department of Crop and Soil Sciences, Washington State University, Pullman, WA 99163, USA; 3 Beijing Institute of Genomics, Chinese Academy of Sciences, Beijing 100101, China; liangyuan@big.ac.cn; 4 China National GeneBank, Environmental Genomics, BGI, Shenzhen 518083, China; yangchentao@genomics.cn

**Keywords:** Bailinggu, comparative transcriptomic analysis, cold stress, qPCR-PCR, EST-SSR

## Abstract

Cold stimulation of Bailinggu’s mycelia is the main factor that triggers primordia initiation for successful production of fruiting bodies under commercial cultivation. Yet, the molecular-level mechanisms involved in mycelia response to cold stimulation are still unclear. Here, we performed comparative transcriptomic analysis using RNA-Seq technology to better understand the gene expression regulation during different temporal stages of cold stimulation in Bailinggu. A total of 21,558 Bailinggu mycelia unigenes were *de novo* assembled and annotated from four libraries (control at 25 °C, plus cold stimulation treatments at −3 °C for a duration of 1–2 days, 5–6 days, and 9–10 days). GO and KEGG pathway analysis indicated that functional groups of differentially expressed unigenes associated with cell wall and membrane stabilization, calcium signaling and mitogen-activated protein kinases (MAPK) pathways, and soluble sugars and protein biosynthesis and metabolism pathways play a vital role in Bailinggu’s response to cold stimulation. Six hundred and seven potential EST-based SSRs loci were identified in these unigenes, and 100 EST-SSR primers were randomly selected for validation. The overall polymorphism rate was 92% by using 10 wild strains of Bailinggu. Therefore, these results can serve as a valuable resource for a better understanding of the molecular mechanisms associated with Bailinggu’s response to cold stimulation.

## 1. Introduction

**Figure 1:**
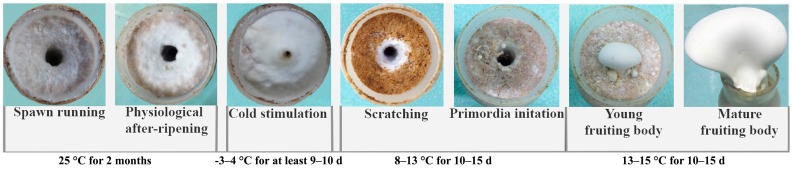
Developmental stages of Bailinggu in year-round mushroom factory cultivation cycles. The vegetative (spawning, physiological ripening, cold stimulation) and fructification (primordia, fruiting body) phases are shown.

*Pleurotus eryngii* subsp. *tuoliensis* [1] is a precious edible mushroom that has been commercially cultivated since 1997 in China [2] and is rapidly growing in popularity throughout Chinese, Japanese, and Korean markets [3,4]. Bailinggu (Figure 1) is the Chinese commercial name for *P. eryngii* subsp. *tuoliensis*. Although yields have steadily increased, many factors still limit more substantial increases in Bailinggu production rates under commercial cultivation [5]. In addition to internal physiological factors that influence mycelia ripening, the cultivation cycle of Bailinggu requires special temperature, humidity, and light conditions to initiate primordia and fruiting bodies [6,7]. Among these environmental factors, cold stimulation of mature mycelia is the main trigger for primordia initiation and plays a crucial role in mushroom formation and subsequent yields of Bailinggu [4,6,7]. For example, without cold stimulation at −3 to 4 °C for 5–6 days (d) following physiological after-ripening of mature mycelia, primordia formation is limited or nonexistent. Moreover, cold temperature stimulation of mature mycelia for 9–10 d have the potential to achieve greater consistency in culture bottle budding of primordia and to ensure a high degree of fruiting body uniformity. However, commercial-scale, artificial cold stimulation is expensive and energy intensive. One potential strategy for increasing production while reducing costs and conserving energy is to shorten the duration of cultivation cycles and cold stimulation treatments. To evaluate the feasibility of this strategy, additional studies are needed to better understand the molecular and physiological mechanisms for Bailinggu growth in response to cold stimulation.

Cold stimulation of Bailinggu includes chilling (0–4 °C) and freezing (<0 °C) temperatures. Previous research has shown that cold temperature treatment of mature mycelia in Bailinggu following physiological after-ripening triggers a series of physiological and biochemical responses [6,7,8]. For example, cold temperatures activate antioxidative enzymes and other enzymes (e.g., protease, laccase, and amylase) that facilitate rapid conversion of accumulated proteins and polysaccharides to new proteins and soluble sugars [8]. Thus, cold stimulation initiates physiological mechanisms that can create the material conditions for primordia differentiation. 

Plants and fungi respond to low temperatures by altering the expression of thousands of genes, thereby changing cellular, physiological, and biochemical processes [9,10,11,12]. Thus, analysis of gene expression would be a valuable tool to understand the transcriptome dynamics and the potential for manipulation of the gene expression pattern in mushrooms. Bailinggu’s genes and their functions associated with energy metabolism, material conversion, and cell growth during cold stimulation have not been studied. Therefore, the molecular-level mechanisms involved in cold temperature gene regulation and signal transduction are still unclear. 

With advances in high-throughput sequencing technologies, RNA sequencing (RNA-seq) technology has been successfully used for gene expression profiling and other transcriptome studies in some edible mushrooms, including *Agaricus bisporus* [13], *Lentinula edodes* [14], *Flammulina velutipes* [15], and *Laccaria bicolor* [16]. In addition, RNA-seq has been applied to quantify RNA levels under chilling stress in edible mushrooms such as *Pleurotus ostreatus* [9], *Tuber melanosporum* [10] and under cold stress in other species such as *Solanum habrochaites* [17], *Corylus heterophylla* [18], *Lilium lancifolium* [19], and *Vaccinium* spp. [20]. Furthermore, the Solexa/Illumina (Illumina) platform has made it possible to perform *de novo* transcriptome sequencing for species without a sequenced genome such as Bailinggu [21,22,23,24,25]. However, no studies have performed a comparative transcriptomic analysis using next-generation sequencing technologies on Bailinggu during cold stimulation.

Transcriptome sequencing is an efficient, cost-effective way to develop transcript/EST-based simple sequence repeats (EST-SSR) markers, which are important resources for genetic diversity analysis, genetic map construction, and molecular marker-assisted selection in breeding [20,24,26]. Transcriptomic sequencing for SSR mining has been used in a wide range of fungal species and plants, including *Auricularia polytricha* [22], *Agaricus subrufescens* [27], *Caragana korshinskii* [25], and *Myrica rubra* [28]. However, SSR and EST-SSR molecular markers have yet to be developed for genetic diversity and mapping studies of Bailinggu.

In this study, we used RNA-seq technology to perform an analysis of Bailinggu mycelia transcriptome under the physiological after-ripening stage and several temporal stages of cold stimulation. Our specific objectives were the following: (1) identify relevant functional gene groups and signaling pathways associated with cold stimulation and (2) screen and identify the EST-SSR markers for ongoing genetic diversity and mapping studies of Bailinggu.

## 2. Results and Discussion

### 2.1. RNA-Seq and De Novo Assembly

Overall, 78.64 million high-quality, clean 100-bp reads were generated from the four libraries, divided into 18.83, 13.80, 27.14 and 18.86 million from the control (the samples were stored at 25 °C), 1–2 d (the samples were stored at −3 °C), 5–6 d (the samples were stored at −3 °C) and 9–10 d (the samples were stored at −3 °C), respectively. The overall GC content was over 51% in both the control and cold stage samples (Table 1).

*De novo* assembly with the clean reads resulted in 21,558 unigenes with an average length of 1248 bp and an N_50_ of 2266 bp (Table 2). Our accuracy check showed that over 88% clean reads mapped to these unigenes, which were then used for further analysis (Table 3). 

Recent studies indicated that the overlapping RNA-seq method is a useful tool for identifying differential gene expression (DEG) in libraries that represent different points in time [29]. We performed the overlapping RNA-seq on four libraries (Control, 1–2 d, 5–6 d, and 9–10 d) to better elucidate year-round cultivation at different temporal stages of cold stimulation. Therefore, the four library datasets generated represent a comprehensive view of Bailinggu response to temperature changes and the cold stimulation process.

### 2.2. Transcriptome Annotation and Functional Classification

**Figure 2:**
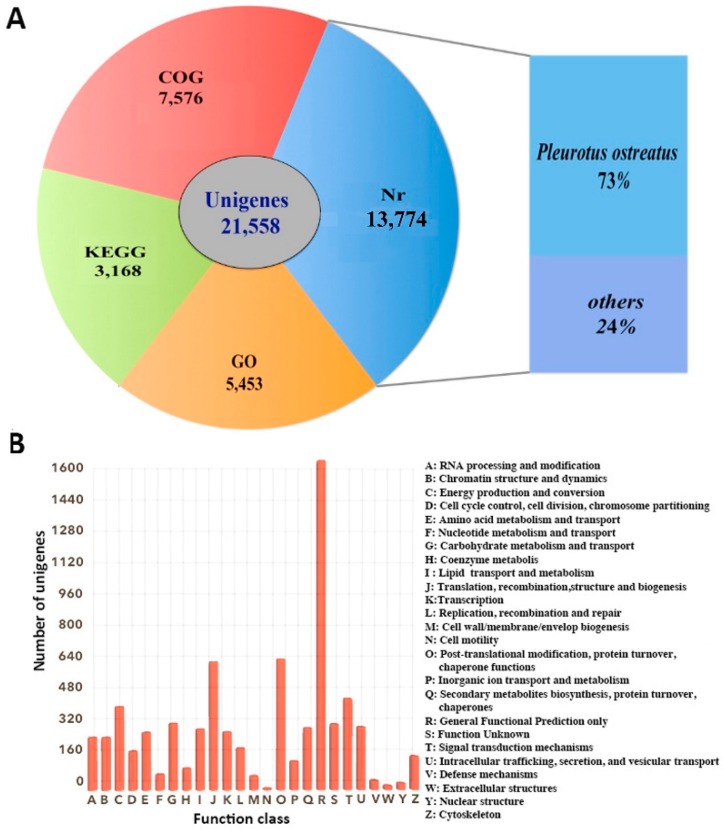
Unigene annotation. (**A**) Number of unigenes blasted to Non-Redundant (NR), Clusters of Orthologous Groups (COG), Gene Ontology (GO) and Kyoto Encyclopedia of Genes and Genomes (KEGG) databases (*e* < 0.00001); (**B**) COG classification. A total of 7576 unigenes were assigned to 25 classifications. The capital letters on the x-axis indicate the COG categories as listed on the right of the histogram.

Annotation and functional classification results of 21,558 unigenes were obtained using the five datasets (Figure 2A). A total of 13,774 (63.89%) unigenes showed hitting with known proteins in the NR database, followed by Interproscan database (11,334, 52.57%), COG database (7576, 35.14%), GO database (5453, 25.29%), and KEGG database (3168, 14.70%). Distribution analysis based on BLASTx searches showed that 73% of the annotated unigenes have homologs in *Pleurotus ostreatus*, which is the species with a sequenced genome most closely related to *P. eryngii* subsp. *tuoliensis* (Bailinggu). COG results showed that a total of 7576 unigenes were assigned to 25 classifications (Figure 2B). The top five of COG categories were associated with (C) Energy production and conversion, (G) Carbohydrate metabolism and transport, (J) Translation, recombination, structure and biogenesis, (O) Post-translational modification, protein turnover, chaperone functions and (T) Signal transduction mechanisms.

### 2.3. Identification of DEGs Involved in Different Stages of Cold Stimulation

**Figure 3:**
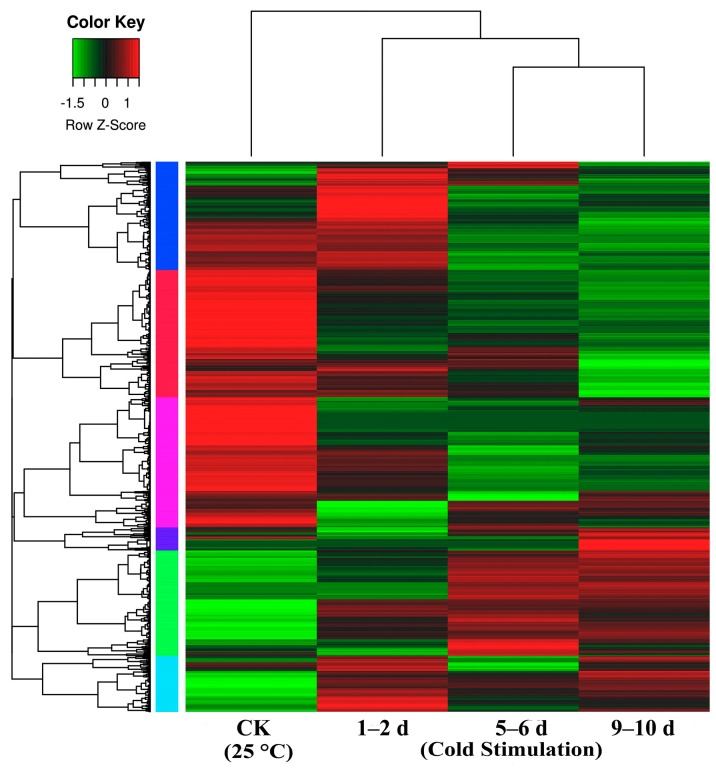
Heat map representation of cluster analysis for the expression patterns of over 7000 differential gene expressions (DEGs) (*p* value: < 0.05, fold change: ≥ 2 fold (log2 |FC > 1|)) between control (CK = 25 °C) and different stages of cold stimulation (−3 °C for a duration of 1–2 d, 5–6 d, and 9–10 d) using the FPKM. The samples of the three stages of cold stimulation cluster together; notably, the 5–6 d and 9–10 d samples cluster particularly close.

We compared libraries for gene expression changes that were ≥2-fold (Figure 3, Table S1). Overall, we detected 2799, 3312, and 2945 up-regulated DEGs and 2504, 3063, and 3091 down-regulated DEGs between the control and each of the cold stage libraries (1–2 d, 5–6 d, and 9–10 d) (Table S1), respectively. Additionally, we detected 2493, and 1997 up-regulated DEGs and 2392 and 2446 down-regulated DEGs between the 1–2 d and 5–6 d libraries and the 5–6 d and 9–10 d libraries, respectively.

Zampieri *et al.* (2011) [10] demonstrated that DEGs listed as environmental stress-responsive genes might help *Tuber melanosporum* adapt to cold temperatures that regulate fructification. The WEGO and KEGG results for our four libraries showed that DEGs listed as responsive to cold stimulation were involved in cell wall and membrane system stabilization, calcium signaling, osmotic regulation, antioxidant enzymatic defense system, and soluble sugars and protein biosynthesis and metabolism.

### 2.4. Genes Involved in Cell Wall and Membrane System Stabilization

A wide range of studies indicate that the cell wall and membrane systems are the primary site for the action of cold-responsive proteins in fungi and plants [23,30,31]. We found unigenes with up- or down-regulated expression during the cold stimulation process that associated with WEGO terms related to “cell wall” and “membrane”. These changes in cell wall and membrane proteins during cold stimulation could be important to maintain cell wall integrity. These associations included unigenes encoding for hydrophobin (TR9892|c2_g1), cell wall-associated hydrolase (TR7761|c1_g2), chitin synthase (TR4601|c1_g4) and plasma membrane proteolipid 3 (TR3136|c0_g1 and TR8280|c0_g1). Hydrophobins are proteins unique to fungi and are a type of cell wall protein [32,33,34] involved in fungal growth and development [35,36,37]; response to environmental factors such as light, nutrient availability, and high temperatures [38]; and resistance to pathogens [39]. We found that unigene (TR9892|c2_g1) encoding for the PoH2-type hydrophobin [40] exhibited up-regulated expression during the entire cold stimulation process. We suggest that PoH2-type hydrophobin in Bailinggu might play important roles associated with cold stimulation and the vegetative mycelium development.

Cold tolerance in fungi and plants requires lipid remodeling at membrane system and gene encoding phospholipase and fatty acid desaturases have the potential to alter phospholipid and lipids composition [41,42,43,44]. We found up-regulated unigenes of delta 9-fatty acid desaturase (TR7433|c4_g1) and phospholipase D (TR7300|c4_g1) between the control and the cold stage libraries. Previous studies have demonstrated that the delta 9-fatty acid desaturase gene (in *Saccharomyces cerevisiae* and *Thetrahymena thermophila*) and the phospholipase gene contribute to chilling or cold tolerance by altering phospholipid and lipid composition [45,46,47,48]. Similarly, our results indicate that changes in the composition of membrane phospholipid and lipids during the cold stimulation process in Bailinggu may function to stabilize membrane structure.

### 2.5. Genes Involved in Calcium Signaling and Osmotic Regulation

As a secondary messenger, Ca^2+^ is required for cold-induced gene expression in fungi and plants and may activate a variety of signaling pathways during cold stress [49,50,51,52]. We found that unigenes encoding calcium sensor and receptor (TR6457|c2_g2, TR7548|c1_g1, TR7021|c7_g1, TR6437|c1_g1 and TR7554|c2_g3) such as calcium-dependent protein kinase and two-component histidine kinase were expressed at higher levels during the cold stimulation stages. These genes have also been associated with cold temperature response in previous studies [21,23,53,54] and, therefore, are likely part of the cold stimulation signal transduction pathways in Bailinggu. 

Mitogen-activated protein kinases (MAPK) pathways may be triggered by receptors/sensors such as histidine kinases under various abiotic stress stimuli, including cold stress in plants and fungi [55,56,57,58,59]. We found that unigenes encoding MAPK kinase (TR7227|c5_g1) and MAPK kinase kinase (TR4680|c1_g1) in the MAPK signaling pathways (ko04011) respond to cold stimulation. Moreover, we found down-regulated unigene expression of tyrosine phosphatase (TR5178|c3_g1) between control and the cold stimulation stages. Lee and Esselman (2002) [60] suggested that the reduced tyrosine phosphatase activity leads to an increase in the output of MAPK pathways. Our results suggest a similar calcium/calmodulin-triggering mechanism in Bailinggu in response to cold stimulation can regulate the expression and activity of kinases in the MAPK pathway. We indicate that Hog1-type MAPK in Bailinggu associated with osmotic regulation during the cold stimulation.

### 2.6. Genes Involved in Antioxidant Enzymatic Defense System

The antioxidant enzymatic defense system and the organic osmolytes are closely related to plant and fungi cold tolerance [9,19]. We found that unigenes encoding enzymes associated with antioxidative enzymes were expressed at higher levels in the cold stimulation stages of Bailinggu. For example, unigenes encoding catalase (TR3796|c0_g1) and peroxidase (TR7183|c0_g1) exhibited higher expression levels in the 5–6 d cold stimulation library. In addition, our data analysis revealed down-regulated gene expression of delta-1-pyrroline-5-carboxylate dehydrogenase (P5CDH, TR7530|c1_g1), which is involved in proline catabolism [61,62] during the cold stimulation process. When P5CDH activity is limited, the P5C-proline cycle can transfer more electrons to the mitochondrial electron transport chain and generate reactive oxygen species (ROS) [63]. Under cold stress, the accumulation of ROS leads to activation of the antioxidant enzyme defense system [20,63]. These results suggest that the antioxidant enzymatic defense system and proline metabolism are positively correlated with detoxification of ROS caused by cold stimulation of Bailinggu.

### 2.7. Genes Involved in Soluble Sugars and Protein Biosynthesis and Metabolism

Using physiological, biochemical, and growth tests on mature mycelia during cold stimulation, we identified abundant conversion of biomacromolecules and biosynthesis of new compounds such as glycogen breakdown and protein synthesis and metabolism [7]. Our KEGG analysis found that the most abundant unigenes were up- or down-regulated expressions involved in the “starch and sucrose metabolism” (ko00500), “glycolysis/gluconeogenesis” (ko00010), “amino sugar and nucleotide sugar metabolism” (ko00520), and “protein processing in endoplasmic reticulum” (ko04141) pathways during cold stimulation. 

The accumulation of soluble sugars has been associated with adaption to cold stress in fungi and plants [21,64,65]. In “starch and sucrose metabolism”, “glycolysis” and “amino sugar and nucleotide sugar metabolism” pathways, we observed unigenes relating to trehalose, glucose, and fructose biosynthesis. The DEGs include unigenes encoding starch 1,4-α-glucan branching enzyme (glycoside hydrolase family 13 protein, TR6876|c2_g1), trehalose 6-phosphate synthase (glycosyltransferase family 20 protein, TR6743|c2_g1), glucan 1,3-beta-glucosidase (glycoside hydrolase family 5 protein, TR6979|c4_g1), glycoside hydrolase family 16 protein (TR1573|c0_g1), glycoside hydrolase family 47 protein (TR3403|c1_g1), and other different glycosyl hydrolase families. Ramírez *et al.* (2011) [9] revealed that the above genes encoding for carbohydrate active enzymes of *Pleurotus ostreatus* were involved in cold stress. These data suggest that the transformation and combination of soluble sugars might be necessary for cold tolerance and energy preservation in Bailinggu during cold stimulation.

Moreover, the cold-responsive genes encoding molecular chaperones, including heat shock proteins Hsp20 (TR2339|c0_g1), Hsp70 (TR2377|c0_g1), and Hsp90 (TR5261|c1_g1) were identified in the “protein processing in endoplasmic reticulum” pathway between the control and the cold stage libraries. These genes were also induced in black truffle, *S. cerevisiae,* and other plants in response to cold stress [10,31,65,66]. This result suggests that the induction of various heat shock proteins might be necessary for fungi and plants to adapt to 4 °C and cold temperatures by stabilizing proteins against cold-induced denaturation. 

Overall, the above results provide evidence to support that carbohydrate metabolism plays the vital role during the cold stimulation process of Bailinggu.

### 2.8. Computational Identification and Prediction of Transcription Factor

We identified 37 TF families from the FTFD and Plant TFDB pipeline (Table S2). TFs comprised the major TF families in most fungi and plants such as, Myb, bHLH, zinc fingers, WRKY, bZIP, and homebox, and the special families in most fungi such as Zn2Cys6 and HMG TFs. 

The TFs controlling the expression of cold-induced genes that increase fungi and plant cold tolerance have been identified [67,68]. In this study, many TFs that play a role in cold stress response were identified, including C3H zinc finger, C2H2 zinc finger, GATA zinc finger, Myb, bZIP, bHLH, WRKY, AP2, NAC, ERF, and RAV TFs. Previous studies also demonstrated that these TF families play diverse roles in fungi and plant developmental processes and environmental responses such as chilling and cold stress resistance [14,22,62,69,70,71]. For example, Myb, bHLH, C2H2 zinc finger and WRKY motifs affect seed germination and growth and induce the enhancement of cold tolerance in higher plants [72,73,74,75,76]. Together, these results might indicate that Myb, bHLH, C2H2 zinc finger and WRKY are also important players in Bailinggu response to cold stimulation.

### 2.9. Validation of Transcriptome Data by qRT-PCR

**Figure 4:**
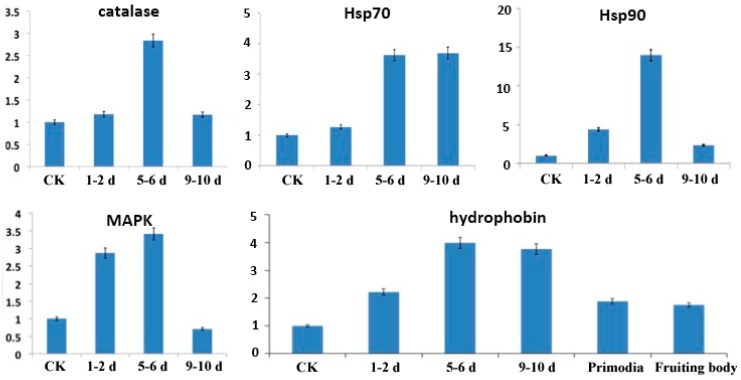
qRT-PCR analysis of selected genes in mycelia during the physiological after-ripening stage (control (CK) = 25 °C) and different cold stimulation stages (1–2 d, 5–6 d, and 9–10 d at −3 °C). Transcript levels of five genes (PoH2-type hydrophobin, MAPK (mitogen-activated protein kinase hog1), catalase, Hsp70 (heat shock protein 70) and Hsp90 (heat shock protein 90)) that appeared to be up-regulated in all cold stimulation stages compared to the physiological after-ripening stage. The X-axis represents relative quantification of transcript levels of the three selected genes, and the Y-axis represents the control and the three sampling times after cold stimulation.

To validate the results of the RNA-seq analysis, five genes were selected to confirm differential expression by quantitative real-time PCR (qRT-PCR). These five genes—PoH2-type hydrophobin, MAPK (mitogen-activated protein kinase hog1), catalase, Hsp70 (heat shock protein 70) and Hsp90 (heat shock protein 90)—are known to relate to cold stress. These genes showed expression patterns similar to the differential analysis results from the RNA-seq output of the bioinformation analysis (Figure 4). That is, all five genes have higher expression levels in the cold stimulation stages compared to the physiological after-ripening stage, but have varying levels of expression among the different cold stages. Genes showing significant differential expression between different stages of cold stimulation can be further explored as candidate genes for cold tolerance using functional genomics approaches.

Overall, all above results suggest that *de novo* transcriptome sequencing for identifying cold-responsive genes in Bailinggu provides a good estimation of gene expression trends and levels in response to temporal variation in cold stimulation conditions. Our methods and results could be helpful in further studies of Bailinggu or in similar studies in the context of other moshrooms.

### 2.10. SSR Mining and Identification

Bailinggu’s genome has not been sequenced until now. So, our transcript/EST-based molecular markers are an important resource for genetic diversity analysis, genetic map construction, and molecular marker-assisted selection in breeding. Overall, 607 transcripts/EST-SSR loci were identified and primers were developed (Table S3). Among them, TNR is the most commonly repeated motif with a frequency of 63.76%, followed by DNR (30.64%), TTNR (3.79%), PNR (1.32%) and HNR (0.49%). TNR has generally been detected with the highest frequency in mushroom and crops, including *Pleurotus ostreatus*, *Boletus edulis*, *Coprinopsis cinerea*, *Schizophyllum commune* [77], *Auricularia polytricha* [22], *Lentinula edodes* [78], maize, rice [79], peanut [80], and *Gossypium hirsutum* [81].

**Figure 5:**
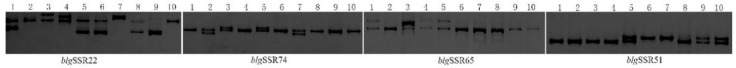
Amplification products obtained from PCR using EST-SSR primers and DNA from 10 Bailinggu wild strains using non-denaturing PAGE. EST-SSR primers are *blg*SSR22, *blg*SSR51, *blg*SSR65, and *blg*SSR74. Lines 1–10 are wild Bailinggu strains collected from Xinjiang Autonomous Region in China.

To obtain high-quality EST-SSR primer pairs and test their polymorphism, we selected 100 primer pairs including 22 DNR, 68 TNR, 8 TTNR, and 2 PNR. Ninety-five out of the 100 SSR primer pairs generated a product in at least one of the tested wild collected strains. Ninety-two primer pairs resulted in polymorphic products for all genotypes of wild collected strains and were distinguishable from each other. The results of five-selected primer pairs used for polymorphism analysis were shown in Figure 5. The TNR primer pairs were the most abundant polymorphism of these primer pairs. Five primer pairs—1 TNR, 3 DNR, and 1 TTNR—failed to generate a product in all tested genotypes. Therefore, the overall amplification rate was 95% and the polymorphism rate was 92%. These 92 primers can be used for subsequent population genetics diversity, genetic linkage, and QTL analysis of agronomic traits for Bailinggu.

## 3. Methods

### 3.1. Mushroom Tissue Source

The commercial strain and mycelia samples of *Pleurotus eryngii* subsp. *tuoliensis* used in this work were kindly provided by Hengdaxing, a year-round cultivation mushroom factory in Beijing, China. Mycelium was grown at 25 °C in cultivation bottles containing 780 g medium (25% corncob, 35% sawdust, 24% wheat bran, 10% maize powder, 4.5% soybean meal) in the dark for 60 days up to the physiological after-ripening stage, which time the substrate was fully colonized in the bottles. Then, the mature mycelia are grown at −3 °C for 1, 2, 5, 6, 9 and 10 d. We randomly selected mature mycelia samples from the different cultivation stages and divided them into two groups, the control sample (the physiological after-ripening stage grown at 25 °C for 60 d) and the plus cold stage samples that were grown at −2–3 °C for 1, 2, 5, 6, 9 and 10 d. All samples were collected and stored in liquid nitrogen in separate bottles and labeled according to number of days under 25 °C and cold stress.

### 3.2. Library Preparation and RNA-Seq

For comparative transcriptomic analysis, we selected 3 bottles from 7 stages respectively, including the control sample (the physiological after-ripening stage grown at 25 °C for 60 d) and the cold stage samples that were grown at −3 °C for 1, 2, 5, 6, 9 and 10 d. Total RNA was extracted from mycelia obtained from each bottles, then equal amounts total RNA from each bottles of one stage mix together. When referring to the 1–2, 5–6 and 9–10 days, equal amounts total RNA obtained from 1 d and 2 d cold samples were merged to represent the first stage of cold stimulation (1–2 d). At the same, the 5 and 6 d cold mycelia samples were merged to represent the second cold stage that the minimum number of days required for primordia initiation (5–6 d). The 9 and 10 d cold mycelia samples were merged to represent the third cold stage (9–10 d) that the minimum number of days required for budding consistency and high production in factory-based Bailinggu cultivation. 

Using TRIzol reagent (Life technologies, New York, NY, USA), total RNA was extracted from mycelia obtained from six culture bottles each for the control and the three cold stage samples. The total RNA was further evaluated for integrity and quality using an Agilent Technologies 2100 Bioanalyzer (Santa Clara, CA, USA). The four cDNA libraries were constructed and a paired-end sequencing strategy (on a Illumina HiSeq 2000 platform) was performed by Beijing Institute of Genomics, Chinese Academy of Sicences (Beijing, China) using the manufacturer’s standard protocol. Data have been deposited in the National Center for Biotechnology Information (NCBI) database under the accession number SRR2080100.

### 3.3. De novo Transcriptome Assembly and Homology Search

Using an in-house Perl script, we first filtered and trimmed the adapters, low-quality sequences, and duplicate sequences of the raw reads to obtain clean data. Then, using Trinity’s [82] standard protocol (http://trinityrnaseq.sf.net), all clean reads from the four libraries were *de novo* assembled to yield transcripts and unigenes.

These genes were assigned putative gene descriptions following Basic Local Alignment Search Tool X (BLASTX) alignment to the Non-Redundant (NR) protein database of NCBI, Gene Ontology (GO) (http://wego.genomics.org.cn) database, Interproscan database, Clusters of Orthologous Groups (COGs) in the COG database (http://www.ncbi.nlm.nih.gov/COG) [82], and Kyoto Encyclopedia of Genes and Genomes (KEGG; http://www.genome.jp/kegg) pathway annotations.

### 3.4. Identification of Differentially Expressed Genes

Differential gene expression (DEG) analysis was calculated using RSEM (RNA-Seq by Expectation Maximization) [83] and R package EBseq [84] and DEGseq [85] to compare the control and the three cold stage libraries. Functional analysis of the differentially expressed genes (DEGs) was carried out using Web Gene Ontology Annotation Plotting (WEGO) [86] and KEGG pathways. The significance of gene expression differences was assessed using the threshold of false discovery rate *p*-value ≤ 0.05 and |log2 ratio| ≥ 1.

### 3.5. Identification of Transcription Factors

Transcription factors (TFs) were identified from the lists of significant DEGs using InterPro terms for conserved domains via the pipeline of Fungal Transcription Factor Database (FTFD) (http://ftfd.snu.ac.kr/) and Plant Transcription Factor Database (Plant TFDB) (http://planttfdb.cbi.pku.edu.cn/) with an E-value cut-off of <10^−5^ [87,88]. The TFs were then used to classify DEGs according to the gene family information. TFs believed to be associated with cold stimulation were selected for further investigation.

### 3.6. Quantitative Real Time PCR

The quantitative real time PCR (qRT-PCR) was used to assess the results of RNA-seq analysis. Samples of mature mycelia from the different cold stages (1–2 d, 5–6 d and 9–10 d at −3 °C) and control (untreated, at 25 °C) were randomly selected from Hengdaxing mushroom factory in Beijing. Total RNA extraction from these samples used the same procedures described above. Approximately 1 μg of total RNA of each sample was subjected to reverse transcription using a RevertAid First Strand cDNA Synthesis Kit (Thermo Scientific, Waltham, MA, USA). The qRT-PCR was performed with SYBR^®^ Green Supermix (Thermo Scientific) on the Stratagene Mx3005P (Agilent Technologies) thermal cycler. Each reaction contained 1 μL of the first-strand cDNA as a template and 10 μM of each primer in a total volume reaction of 20 μL. The amplification program was performed under the following conditions: 95 °C for 30 s, followed by 40 cycles of 95 °C for 5 s and 60 °C for 30 s. The gene-specific primers were used for qRT-PCR are shown in Table S4. Two biological and three technical repeats were performed for each sample. The actin gene (GenBank accession number: AY772706) was used as an internal reference gene for normalization of data. Relative gene expression level was calculated using the 2^−ΔΔCt^ method [89].

### 3.7. SSR Mining and Identification

The MIcroSAtellite (MISA) identification tool [90] was utilized to identify simple sequence repeats of the final genes. Criteria of default parameters for SSR primer development were dinucleotide repeats (DNR) ≥ 6, trinucleotide repeats (TNR) of ≥ 5, tetranucleotide repeats (TTNR) ≥ 5, pentanucleotide repeats (PNR) ≥ 5, and hexanucleotide repeats (HNR) ≥ 5. Primers were generated from the primer modelling software Primer3 (Version 2.3.5, Whitehead Institute, Cambridge, MA, USA). Then we removed the same potential SSR loci and the primers of the different isoforms of the same gene.

One hundred SSR primers were tested for amplification and polymorphism of Bailinggu. Ten wild collected strains of Bailinggu collected from Xinjiang Autonomous Region of China were evaluated in this work. All strains (NO. CCMJ2501-2510) are maintained in the Engineering Research Center of Chinese Ministry of Education for Edible and Medicinal Fungi of the Jilin Agriculture University, China. Genomic DNA was isolated from mycelia using the Plant DNA Mini Kit (KANGWEI, Beijing, China) following manufacturer instructions. Quality of isolated genomic DNA was assessed by the NanoDrop 2000c spectrophotometer (Thermo Scientific). Fifty SSR primers were synthesized at Sangon Biotech Co., Ltd. (Shanghai, China). PCR amplification reactions (20 μL total volume) contained 1 × buffer (Mg^2+^ free), 2 mM MgCl_2_, 0.2 mM dNTPs, 0.2 μM primers, 0.5 units of DNA Polymerase (Thermo Scientific), and 1.0 ng genomic DNA. PCR was performed as follows: denaturation at 94 °C for 5 min, followed by 30 cycles of 94 °C for 30 s, 60 °C for 30 s, 72 °C for 30 s, and a final step at 72 °C for 8 min. PCR products were mixed with a volume of loading buffer and then subjected to 8% polyacrylamide gel for 1.5 hours.

## 4. Conclusions

In this study, we presented comprehensive profiles of the transcriptome of *Pleurotus eryngii* subsp. *tuoliensis* (in the mycelium tissue) and their changes during the cold stimulation process using RNA-Seq. By comparing different gene expression profiles, we revealed candidate genes and complex regulatory networks that play key roles in signal transduction, cell growth, and metabolite biosynthesis in response to cold stimulation. A large number of genes involved in diverse biological or molecular pathways were identified during the cold stimulation process, including the following: (1) genes involved in cold signal sensors or transduction; (2) genes encoding cold-regulated proteins associated with cell wall and membrane; (3) antioxidant enzymatic defense system genes; (4) genes associated with soluble sugars and protein biosynthesis and metabolism; and (5) stress-responsive transcription factor genes. Our results also demonstrated that a series of complex regulatory networks are triggered in Bailinggu during cold stimulation process. Our study provides new insights into the molecular mechanisms regulating Bailinggu mycelium tissue response to cold stimulation. Our study can also serve as a valuable resource for future, relevant genetic research associated with cold stimulation in edible mushrooms. Furthermore, numerous SSR loci were predicted based on transcripts/EST and 607 SSR primers were designed; 92 out of the 100 detected SSR primer pairs generated a product in at least one of the tested wild collected strains. These loci and primers can be used for subsequent population genetics diversity, genetic linkage, and QTL analysis of agronomic traits for Bailinggu.

## Figures and Tables

**Table 1:** Basic statistics of RNA-seq reads obtained from Illumina HiSeq-2000.

	CK (25 °C)	1–2 d (−3 °C)	5–6 d (−3 °C)	9–10 d (−3 °C)
Rawdata-reads	21,719,764	19,961,572	32,377,356	25,025,308
Rawdata(bp)	2,193,696,164	2,016,118,772	3,270,112,956	2,527,556,108
Clean-reads	18,830,230	13,801,100	27,140,932	18,863,168
Cleandata(bp)	1,888,917,231	1,372,420,836	2,723,972,886	1,891,155,067
Cleanreads-GC (%)	51.80%	51.16%	52.32%	51.40%
Cleanreads-Q20	97.09%	93.70%	95.94%	95.98%

Four libraries: control (CK = 25 °C) and different stages of cold stimulation (−3°C for a duration of 1–2 d, 5–6 d, and 9–10 d).

**Table 2:** *De novo* assembly results using Trinity.

>200 bp	N_50_	Number	bp	GC%	Average Length (bp)
Isoforms	2997	49,070	93,081,194	51.46%	1248
Unigenes	2266	21,558	26,907,923	51.05%	1248

**Table 3:** Clean reads mapping to unigenes.

	CK (25°C)	1–2 d (−3 °C)	5–6 d (−3 °C)	9–10 (−3 °C)
Reads	18,830,230	13,801,100	27,140,932	18,863,168
Mapping	17,181,312	12,259,774	25,547,464	17,726,484
Unique	14,572,191	9,912,847	21,082,048	14,721,561
Mapping rate	91.24%	88.83%	94.13%	93.97%
Unique rate	77.39%	71.83%	77.68%	78.04%

Four libraries: control (CK = 25 °C) and different stages of cold stimulation (−3 °C for a duration of 1–2 d, 5–6 d, and 9–10 d).

## Supplementary Materials

Supplementary materials can be accessed at: http://www.mdpi.com/1420-3049/21/5/560/s1.

## Abbreviations

The following abbreviations are used in this manuscript:

DEGs: Differentially expressed genes
MAPK: Mitogen-activated protein kinases
DNR: Dinucleotide repeats
TNR: Trinucleotide repeats
TTNR: Tetranucleotide repeats
PNR: Pentanucleotide repeats
HNR: Hexanucleotide repeats
MISA: MIcroSAtellite
TFs: Transcription factors
qRT-PCR: Quantitative real time PCR
P5CDH: δ-1-Pyrroline-5-carboxylate dehydrogenase
ROS: Reactive oxygen species
